# A benchmarking protocol for intact protein-level Tandem Mass Tag (TMT) labeling for quantitative top-down proteomics^☆^

**DOI:** 10.1016/j.mex.2022.101873

**Published:** 2022-10-07

**Authors:** Yanting Guo, Dahang Yu, Kellye A. Cupp-Sutton, Xiaowen Liu, Si Wu

**Affiliations:** a Department of Chemistry and Biochemistry, University of Oklahoma, 101 Stephenson Parkway, Room 2210, Norman, OK 73019, United States; b John W. Deming Department of Medicine, Tulane University School of Medicine, New Orleans Bioinnovation Center, Room 422, United States

**Keywords:** Isobaric chemical tag, TMT, Protein-level TMT labeling, Top-down proteomics, Quantification

## Abstract

Isobaric chemical tag labeling for quantification of intact proteins in complex samples is limited due to the tendency of intact proteins precipitate under labeling conditions and increased sample complexity as a result of side products (*i.e.*, incomplete labeling or labeling of unintended residues). To reduce precipitation under labeling conditions, we developed a technique to remove large proteoforms that allowed for the labeling and characterization of small proteoforms (<35 kDa) using top-down proteomics. We also systematically optimized protein-level Tandem Mass Tag (TMT) labeling conditions to obtain optimal labeling parameters for complex samples. Here, we present a benchmarking protocol for protein-level TMT labeling for quantitative top-down proteomics, including complex intact protein sample preparation, protein-level TMT labeling, top-down LC/MS analysis, and TMT reporter ion quantification.

• An optimized protocol for protein-level TMT labeling in complex sample.
• Limits production of incorrectly labeled side products for minimization of spectral complexity.
• A guideline for isobaric chemical tag quantification in top-down proteomics.

## Graphical abstract

**Figure:**
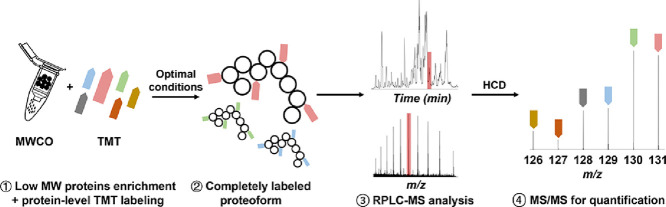

Specification table

**Table:** 

Subject area:	Chemistry
More specific subject area:	Mass spectrometry-based top-down proteomics
Method name:	Protein-level TMT labeling in complex sample
Name and reference of original method:	^1^Yanting Guo, Dahang Yu, Kellye A. Cupp-Sutton, Xiaowen Liu, and Si Wu. “Optimization of Protein-Level Tandem Mass Tag (TMT) Labeling Conditions in Complex Samples with Top-Down Proteomics.” Analytica Chimica Acta (2022): 340037, DOI: 10.1016/j.aca.2022.340037
Resource availability	All python codes are available free of charge in the supplementary file.

## Method details

### *HeLa* cell culture and cell lysate preparation

Detailed procedure is shown in **in Supplementary Document**.

### Protein-level TMT labeling

**1. Materials**
 1.1. Sartorius Vivaspin™ 20 Centrifugal Concentrator (100 kDa MWCO spin filter, Fisher Scientific, Waltham, MA, USA, 14558509).
 1.2. Sartorius Vivaspin™ Turbo 15 PES Centrifugal Concentrator (10 kDa MWCO spin filter, Fisher Scientific, 14558651).
 1.3. Eppendorf Centrifuge 5804 R with S-4-72 rotor (Eppendorf, Enfield, CT, USA).
**2. Chemicals**
 2.1. Triethylammonium bicarbonate (TEAB) buffer (pH = 8.5, Sigma-Aldrich, St. Louis, MO, USA, T7408).
 2.2. TMTsixplex isobaric label reagent (Thermo Fisher Scientific, Waltham, MA, USA, 90061).
 2.3. Urea (Sigma-Aldrich, St. Louis, MO, USA, 208884).
 2.4. Iodoacetamide (IAA) (Sigma-Aldrich, St. Louis, MO, USA, I1149).
 2.5. 0.5 M Tris(2-carboxyethyl) phosphine (TCEP) (Thermo Fisher Scientific, Waltham, MA, USA, 77720).
 2.6. 50% hydroxylamine solution (Sigma-Aldrich, St. Louis, MO, USA, 467804).
 2.7. Pierce™ BCA Protein Assay Kit (Thermo Fisher Scientific, Waltham, MA, USA, 23225).
**3. Procedures**

**Fig. 1:**
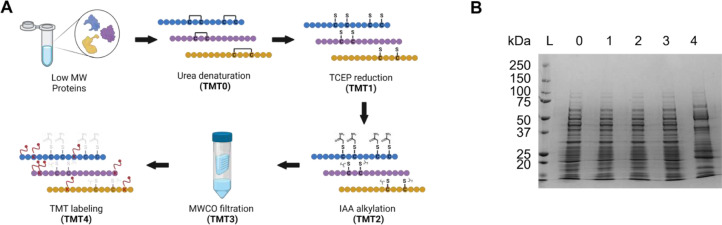
Protein-level TMT labeling sample preparation. (A) Overall complex protein sample preparation workflow. (B) SDS-PAGE analysis of protein samples taken from each step (L: standard protein ladder); TMT0: urea denaturation; TMT1: TCEP reduction; TMT2: IAA alkylation; TMT3: MWCO buffer exchange; TMT4: TMT labeling. Figure was created in BioRender.

The sample preparation workflow for protein-level TMT labeling is shown in Fig. 1A.

 ***3.1. Sample preparation prior to TMT labeling.***
  3.1.1. Rinse spin filters using ∼5 mL labeling buffer (100 mM TEAB, pH = 8.5) through centrifugation at 4200 RPM (3234 × g) and 4 °C for ∼5 min.
  3.1.2. Remove large proteins (>100 kDa) in *HeLa* cell lysate using 100 kDa MWCO filtration through centrifugation at 3234 × g and 4 °C for 20 min. Remove the filtrate and add ∼5 mL of the labeling buffer to wash the proteins and centrifuge at 3234 × gand 4 °C for 20 min. Remove the filtrate again and combine with previous filtrate. Repeat rinsing 3–5 times.
  3.1.3. Combine all the filtrate from Step 3.1.2 and concentrate using a 10 kDa MWCO spin filter through centrifugation at 3234 × g and 4 °C until the protein concentration is ∼ 2 μg/μL. Measure protein concentration using BCA assay.
  3.1.4. Mix the concentrated protein sample (800 μg) with 6 M urea at equal volume for protein denaturation. Final concentration of urea is ∼3 M and protein concentration is ∼1 μg/μL.
   3.1.4.1. Note: Protein mass (800 μg) may be adjusted based on experimental design, but concentration should remain consistent for all samples.
   3.1.4.2. Note: Urea should be prepared fresh.
  3.1.5. Add 160 μL of 0.5 M TCEP to the denatured protein solution (800 μg) to reduce disulfide bonds. Incubate for 15 min at room temperature and then add 215 μL (375 mM) IAA at room temperature. Incubate this solution in the dark for 30 min.
   3.1.5.1. Note: IAA is light sensitive and should be prepared in labeling buffer and wrapped with aluminum foil to prevent decomposition.
  3.1.6. Buffer exchange the alkylated protein solution to 100 mM TEAB buffer (pH = 8.5) and concentrate to ≥1 μg/μL using a 10 kDa MWCO spin filter.
   3.1.6.1. Note: A small portion (∼10 μg) of proteins after each step (urea denaturation, TCEP reduction, IAA alkylation, MWCO buffer exchange) may be removed and analyzed using SDS-PAGE to observe sample consistency (Fig. 1B: Sample 1–4).
 ***3.2. TMT labeling of intact complex protein mixture.***
  3.2.1. Centrifuge TMT reagent tubes for 1 min with a benchtop centrifuge, add 41 μL of ACN to each tube, and vortex the tubes briefly to dissolve TMT. Then centrifuge the tubes briefly using a benchtop centrifuge to bring solution down to the bottom of the tube.
  3.2.2. Mix 50 μg of protein sample produced in Step 3.1.6 (concentration ≥1 μg/μL) with 200 μg TMT reagent in 10 μL ACN. Incubate the sample at room temperature for 1 h, then quench the reaction by addition of 5% hydroxylamine to a final concentration of >0.3% (final pH > 9.1) for 15 min at room temperature.
   3.2.2.1. Note: Protein mass may be adjusted based on experimental design; however, it is recommended that the protein amount be no less than 20 μg for good labeling efficiency.
   3.2.2.2. Note: If the protein concentration is lower than 1 μg/μL, double labeling can be applied. After the first 1 h reaction, add the same amount of TMT reagent (200 μg in 10 μL) to the solution and incubate for 1 additional hour, then quench the reaction using hydroxylamine to a final concentration of >0.3% (final pH > 9.1).
  3.2.3. Analyze TMT-labeled samples by SDS-PAGE to make sure there is no obvious sample loss after TMT labeling (Fig. 1B**: Sample 4**).
  3.2.4. Centrifuge the TMT-labeled sample at 12,000 × g and 4 °C for 30 min before LC-MS/MS analysis to remove any possible precipitation.

### Top-down RPLC-MS/MS analysis

**1. Supplies**
 1.1. Home-packed C5 trapping column (150 μm i.d., 5 cm length, Jupiter particles, 5 μm diameter, 300 Å pore size) [3,4].
 1.2. Home-packed C5 RPLC capillary column (75 μm i.d., 70 cm length, Jupiter particles, 5 μm diameter, 300 Å pore size) [3,4].
 1.3. Modified Thermo Scientific (Waltham, MA, USA.) Accela LC system [5,6].
 1.4. Thermo Orbitrap Exploris 240 mass spectrometer (Thermo Fisher Scientific, Bremen, Germany)
**2. Chemicals**
 2.1. LC-MS grade acetonitrile (ACN) (Sigma-Aldrich, St. Louis, MO, USA, 34998).
 2.2. LC-MS grade iso-propanol (IPA) (Sigma-Aldrich, St. Louis, MO, USA, 34863).
 2.3. Pierce™ Trifluoroacetic Acid (TFA), LC-MS Grade (Thermo Fisher Scientific, Waltham, MA, USA, 85183).
 2.4. Acetic acid, glacial, ACS reagent, ≥99.7% (Sigma-Aldrich, St. Louis, MO, USA, 695092).
 2.5. LC-MS grade HPLC water (Sigma-Aldrich, St. Louis, MO, USA, 270733).
**3. Procedures [** 1 **,** 2 **]**
 3.1. Mobile phase A (MPA) is made up of 0.01% TFA, 0.585% acetic acid, 2.5% IPA, and 5% ACN in water. To prepare 1 L of MPA, add 50 mL ACN, 25 mL IPA, and 920 mL HPLC water to a 1000 mL media storage bottle. Then, add 5.85 mL acetic acid and 0.1 mL TFA to the solution using a glass pipette. Sonicate the buffer for 15 min before use and store at room temperature [7].
  3.1.1. Note: MPA should be prepared in the hood. Gloves, goggles, and lab coat should be worn while preparing buffer.
  3.1.2. Note: Trifluoroacetic acid (TFA) may react with oxygen containing plastics and should only be transferred using glass pipettes. Additionally, TFA is a corrosive chemical that can irritate throat, nose, and lungs and may also burn the skin and eyes. Therefore, proper PPE should be worn while handling TFA.
 3.2. Mobile phase B is made up of 0.01% TFA, 0.585% acetic acid, 45% IPA, and 45% ACN in water. To prepare 1 L of MPB, add 450 mL ACN, 450 mL IPA, and 95 mL HPLC water to a 1000 mL media storage bottle. Then, add 5.85 mL acetic acid and 0.1 mL TFA to the solution using a glass pipette. Sonicate the buffer for 15 min before use and store at room temperature.
  3.2.1. Note: MPB should be prepared in the hood. Gloves, goggles, and lab coat should be worn while preparing buffer.
  3.2.2. Note: Trifluoroacetic acid (TFA) may react with oxygen containing plastics and should only be transferred using glass pipettes. Additionally, TFA is a corrosive chemical that can irritate throat, nose, and lungs and may also burn the skin and eyes. Therefore, proper PPE should be worn while handling TFA.
 3.3. Dilute the TMT-labeled protein sample with HPLC water to a final organic percentage below 10%. Then load 10 μg of the diluted TMT-labeled protein sample onto a home-packed trapping column with a flow rate of 5∼8 μL/min for 20 min.
 3.4. Proteins bound on trapping column are then eluted onto a C5 RPLC capillary column using a modified Thermo Scientific (Waltham, MA, USA.) Accela LC system for seperation [5,8]. A 200-min gradient from 10% to 70% of MPB is applied for protein separation at a flow rate of 400 nL/min. The LC eluent is analyzed by an Orbitrap Exploris 240 mass spectrometer in positive mode (Thermo Fisher Scientific, Bremen, Germany) using a customized nano-ESI interface [3].
  3.4.1. Gradient time can be adjusted according to sample requirements.
 3.5. Parameters used for Orbitrap Exploris 240 mass spectrometer have been previously reported [2]. Briefly, the temperature of the inlet capillary is set to 275°C and the spray voltage is 2.6 kV. The resolution of full MS scans (500–2000 m/z) is set to 120,000 with three micro scans. Top 6 most abundant precursor ions (charge 4–50) are selected for MS/MS fragmentation. The resolution of MS/MS scans (100–1600 m/z) is set to 120,000 with two microscans with 2.0 m/z as the isolation window, and 20 s as the dynamic exclusion. Higher-energy collisional dissociation (HCD) with a normalized energy as 35% is performed for MS/MS fragmentation. The maximum injection time is set to 1000 ms for full mass scans and 500 ms for MS/MS scans. The AGC target is set to 3E6 for full mass scans and 1E6 for MS/MS scans.**Data analysis**
**1. Software**
 1.1. MSconvert [9].
 1.2. TopPIC Suite [10] (includes TopFD and TopPIC).
 1.3. Python 3.8.
 1.4. ProSight Lite [11].
**2. Procedures**
 2.1.***Identification:*** Convert all MS raw files to mzML format using MSconvert [9].
  2.1.1. Set the TopFD [10] deconvolution with parameters as follows: MS1 signal-to-noise ratio as 3, MS2 signal-to-noise as 1, the precursor window size as 3.0 *m/z,* maximum mass as 50,000 Dalton, maximum charge as 30, and m/z error as 0.02. Parameters not given here are default.
  2.1.2. TopPIC [10] is utilized for identification against the annotated Human protein database (UniProt 2021-05-14, 20380 species). Parameters for TopPIC identification are decoy database searching with FDR = 0.01 for spectrum and proteoform level. The maximum number of mass shifts is 2 and the mass shift range is ± 500 Dalton. Input a text file with the PTMs given in Table 1 is used as a fixed modification file. Parameters not given here are default.

**Table 1:** Post-translational modifications (PTMs) in TopPIC Suite searching.

PTM	Monoisotopic mass (Da)	Residues	UnimodID	Status
Acetylation	42.0106	K	1	Variable
Carboxymethyl	58.0055	C	6	Variable
Phosphorylation	79.9663	STY	21	Variable
Oxidation	15.9949	MCPKDNRY	35	Variable
Methylation	14.0157	CKRHDENQ, N-termini	34	Variable
TMT6plex	229.1629	K, N-termini	737	Fixed
 2.2.***TMT Quantitation**:*** To evaluate the protein-level TMT labeling quantification, separate the buffer-exchanged protein sample from Step 3.1.6 to four aliquots and label individually with four tags from TMTsixplex (127, 128, 130, and 131), then mix the TMT-labeled samples with the mass ratio as 5:2:1:1 for LC-MS/MS analysis. Extract the intensities of the reporter ions for each scan from converted mzML files using an in-house python coding described previously [2]. Calculate and compare the normalized reporter ion intensity ratios (127/131, 128/131, 130/131) among samples with the theoretical ratios (5:2:1) (Example in Fig. 2). Here, the observed ratio is 5.00 ± 1.37, 1.97 ± 0.76, 1.05 ± 0.34, which is close to theoretical ratio as 5:2:1) [1], suggesting accurate quantification as expected.

**Fig. 2:**
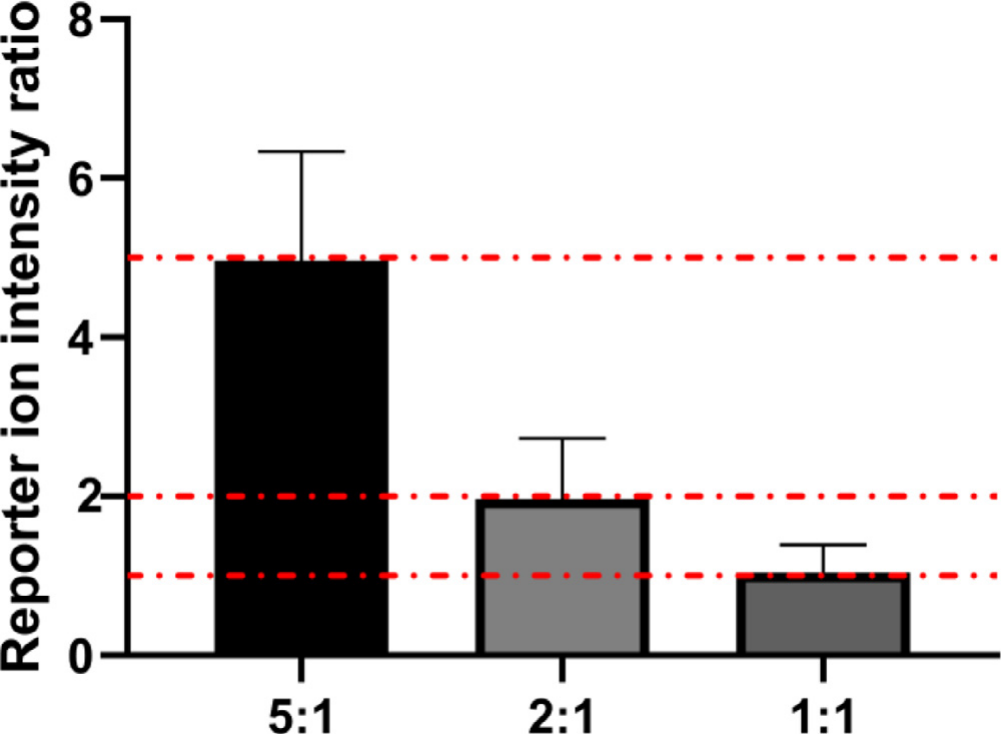
Bar graph shows the normalized intensity ratio of TMT-labeled *HeLa* proteins (127/131, 128/131, and 130/131). The error bars were the calculated standard deviation, and the red dashed lines represent the theoretical ratio of 5:2:1.

**Fig. 3:**
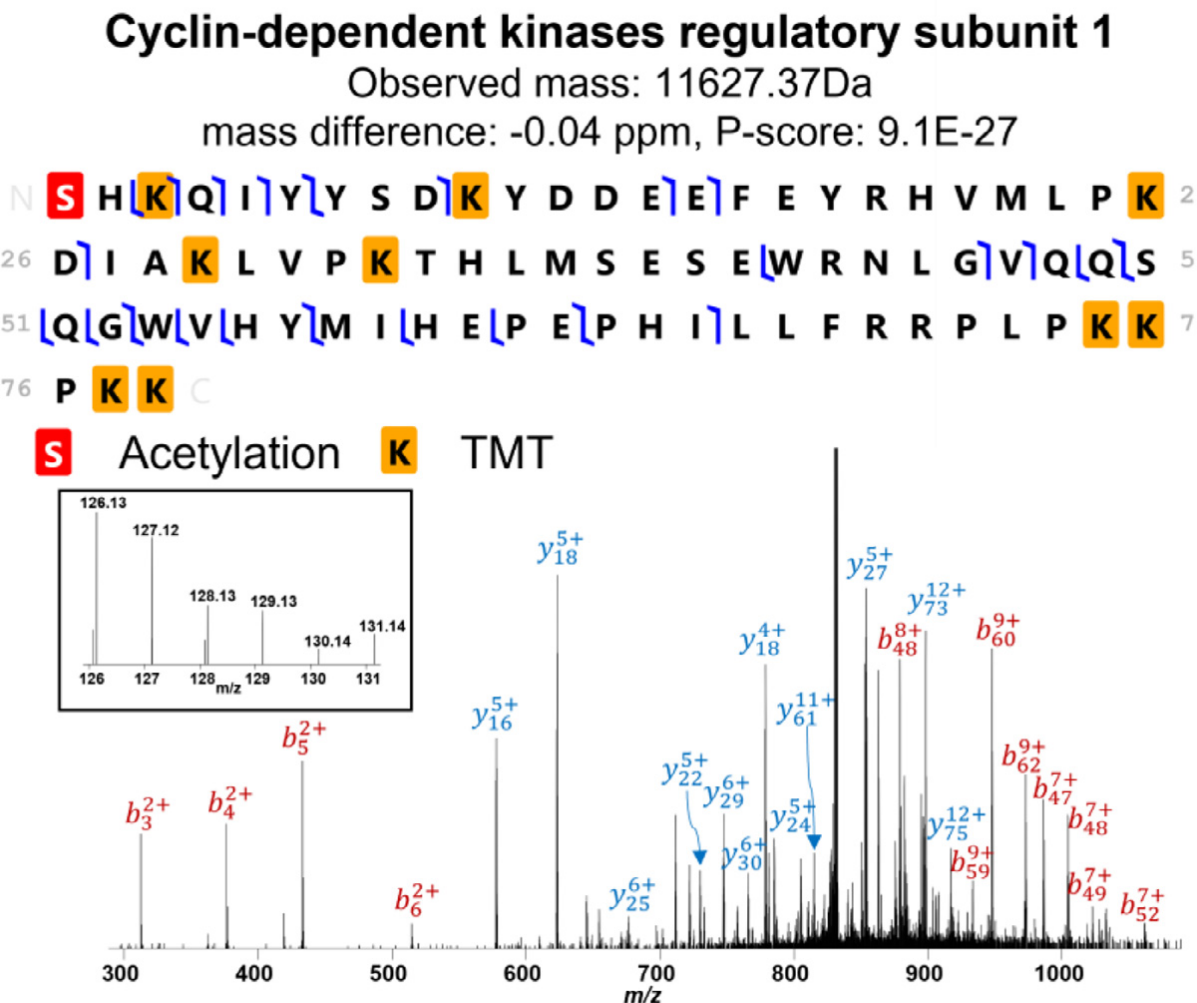
Fragmentation and post-transilational modifications observed on cyclin-dependent kinases regulatory subunit 1 (Uniprot ID: P61024) created by ProSight Lite. Red highlight represents acetylation; yellow highlights represent TMT tags .
 2.3.***Data visualization:*** Protein identification can be confirmed and visualized using ProSight Lite [11]. Load the protein mass list, precursor monoisotopic mass, and sequence obtained for the protein of interest from TopPIC into ProSight to visualize fragmentation. Appropriate PTMs or TMT tags can be manually placed on the amino acid residues to obtain the best fragmentation patterns. As shown in Fig. 3, cyclin-dependent kinases regulatory subunit 1 (Uniprot ID: P61024) from TMT-labeled *HeLa* cell lysate demonstrates there is an acetylation at the N-terminal and a TMT tag on each lysine residue. Quantification result can be obtained from the relative intensity of the reporter ions.

## Declaration of Competing Interest

The authors declare that they have no known competing financial interests or personal relationships that could have appeared to influence the work reported in this paper.

## Data Availability

Data will be made available on request.

## Appendix Supplementary materials

SUPPLEMENTARY INFORMATION **Supplementary Document.doc** Supplementary methods *HeLa* cell culture and cell lysate preparation Python coding for reporter ions extraction for quantification Image, application 1

## References

[1] Guo Y., Yu D., Cupp-Sutton K.A., Liu X., Wu S. (2022). Optimization of protein-level tandem mass tag (TMT) labeling conditions in complex samples with top-down proteomics. Anal. Chim. Acta.

[2] Yu D., Wang Z., Cupp-Sutton K.A., Guo Y., Kou Q., Smith K., Liu X., Wu S. (2021). Quantitative top-down proteomics in complex samples using protein-level tandem mass tag labeling. J. Am. Soc. Mass Spectrom..

[3] Wang Z., Liu X., Muther J., James J.A., Smith K., Wu S. (2019). Top-down mass spectrometry analysis of human serum autoantibody antigen-binding fragments. Sci. Rep..

[4] Wang Z., Ma H., Smith K., Wu S. (2018). Two-dimensional separation using high-PH and low-PH reversed phase liquid chromatography for top-down proteomics. Int. J. Mass Spectrom..

[5] Wu S., Brown R.N., Payne S.H., Meng D., Zhao R., Tolić N., Cao L., Shukla A., Monroe M.E., Moore R.J., Lipton M.S., Paša-Tolić L. (2013). Top-down characterization of the post-translationally modified intact periplasmic proteome from the bacterium novosphingobium aromaticivorans. Int. J. Proteom..

[6] Ansong C., Wu S., Meng D., Liu X., Brewer H.M., Kaiser B.L.D., Nakayasu E.S., Cort J.R., Pevzner P., Smith R.D., Heffron F., Adkins J.N., Paša-Tolić L. (2013). Top-down proteomics reveals a unique protein s-thiolation switch in salmonella typhimurium in response to infection-like conditions. PNAS.

[7] Cupp-Sutton Kellye A., Wang Zhe, Yu Dahang, Wu Si. Proteoform Identification Methods and Protocols Chapter 4: RPLC-RPLC-MS/MS for Proteoform Identification, 1st ed.; 1064–3745, Humana, New York, NY, 2022. 31–42. 10.1007/978-1-0716-2325-1_4 PMC9523456 35657585

[8] Wu S., Yang F., Zhao R., Tolić N., Robinson E.W., Camp D.G., Smith R.D., Paša-Tolić L. (2009). Integrated workflow for characterizing intact phosphoproteins from complex mixtures. Anal. Chem..

[9] Chambers M.C., Maclean B., Burke R., Amodei D., Ruderman D.L., Neumann S., Gatto L., Fischer B., Pratt B., Egertson J., Hoff K., Kessner D., Tasman N., Shulman N., Frewen B., Baker T.A., Brusniak M.Y., Paulse C., Creasy D., Flashner L., Kani K., Moulding C., Seymour S.L., Nuwaysir L.M., Lefebvre B., Kuhlmann F., Roark J., Rainer P., Detlev S., Hemenway T., Huhmer A., Langridge J., Connolly B., Chadick T., Holly K., Eckels J., Deutsch E.W., Moritz R.L., Katz J.E., Agus D.B., MacCoss M., Tabb D.L., Mallick P. (2012). A cross-platform toolkit for mass spectrometry and proteomics. Nat. Biotechnol..

[10] Liu Z., Wang R., Liu J., Sun R., Wang F. (2019). Global quantification of intact proteins via chemical isotope labeling and mass spectrometry. J. Proteome Res..

[11] Fellers R.T., Greer J.B., Early B.P., Yu X., LeDuc R.D., Kelleher N.L., Thomas P.M. (2015). ProSight lite: graphical software to analyze top-down mass spectrometry data. Proteomics.

