# Supplementary material for: A benchmarking protocol for intact protein-level Tandem Mass Tag (TMT) labeling for quantitative top-down proteomics
Source: MethodsX. 2022 Oct 7;9:101873. doi: 10.1016/j.mex.2022.101873 (PMC9587358; doi:10.1016/j.mex.2022.101873)
Supplement: Supplementary file 1 — SUPPLEMENTARY INFORMATION Supplementary Document.doc Supplementary methods HeLa cell culture and cell lysate preparation Python coding for reporter ions extraction for quantification [file mmc1.docx]

*Supplementary information for*

A Benchmarking Protocol for Intact Protein-Level Tandem Mass Tag (TMT) Labeling for Quantitative Top-Down Proteomics

Yanting Guo^1^, Dahang Yu^1^, Kellye A. Cupp-Sutton^1^, Xiaowen Liu^2^, Si Wu^1^*

1. Department of Chemistry and Biochemistry, University of Oklahoma, 101 Stephenson Parkway, Norman, OK 73019

2. John W. Deming Department of Medicine, Tulane University, New Orleans, LA 70112

^*^To whom correspondence should be addressed: Si Wu, Email: si.wu@ou.edu

ABSTRACT

Isobaric chemical tag labeling for quantification of intact proteins in complex samples is limited due to the tendency of intact proteins precipitate under labeling conditions and increased sample complexity as a result of side products (i.e., incomplete labeling or labeling of unintended residues). To reduce precipitation under labeling conditions, we developed a technique to remove large proteoforms that allowed for the labeling and characterization of small proteoforms (< 35 kDa) using top-down proteomics. We also systematically optimized protein-level tandem mass tag (TMT) labeling conditions to obtain optimal labeling parameters for complex samples. Here, we present a benchmarking protocol for protein-level TMT labeling for quantitative top-down proteomics, including complex intact protein sample preparation, protein-level TMT labeling, top-down LC/MS analysis, and TMT reporter ion quantification.

- An optimized protocol for protein-level TMT labeling in complex sample
- Limits production of incorrectly labeled side products for minimization of spectral complexity
- A guideline for isobaric chemical tag quantification in top-down proteomics

**Table of Contents**

[**Supplementary methods** 3](#_Toc103866881)

[***HeLa* cell culture and cell lysate preparation** 3](#_Toc103866882)

[**Supplies** 3](#_Toc103866883)

[**Chemicals** 3](#_Toc103866884)

[**Procedures** 3](#_Toc103866885)

[**Python coding for quantification reporter ions extraction** 6](#_Toc103866886)

[**Reference:** 6](#_Toc103866887)

# **Supplementary methods**

***HeLa* cell culture and cell lysate preparation**^1,2^

### **Supplies**

1. Nunc™ EasYDish™ Dishes (Thermo Fisher, 150466)
2. Sterile pipettes
3. 50 mL falcon tubes (Corning, 430828)
4. Eppendorf Centrifuge 5804 R with rotors S-4-72 and FA-45-45-11

### **Chemicals**

1. *HeLa* cell stock (stored at -80°C)
2. Eagle's minimal essential medium- high glucose (DEME) (Sigma-Aldrich: D6429)
3. Penicillin-streptomycin (Sigma-Aldrich: P4333)
4. Fetal bovine serum (FBS) (Fisher Scientific, SH3008704HI)
5. TrypLE™ Express Enzyme (1X), phenol red (Thermo Fisher Scientific, 12605-010)
6. Dulbecco's Phosphate buffered saline, 10X (Sigma-Aldrich, D1408)
7. 75% ethanol
8. Pierce™ BCA Protein Assay Kit (Thermo Fisher Scientific, Waltham, MA, USA, 23225)

### **Procedures**

*HeLa* cells were grown and lysed as described previously.^1^ *HeLa* cells must be cultured in a biosafety cabinet under sterile conditions. Proper PPE should be worn.

**Step 1: *HeLa* cell recovery**

1. Mix DMEM with pen-strep and FBS to final concentration of 10% FBS and 2% penicillin-streptomycin. Then warm the DMEM mix in a water bath at 37 °C for around 1 hour. Thaw the frozen *HeLa* cells in a 37 °C water bath quickly.
2. Add 8 mL of the DMEM mixture into the cell culture plate, transfer the *HeLa* cells into the plate. Shake the plate gently on the bench.
3. Incubate the cells at 37 °C with 5% CO_2_. Change the media every two days until the cell density is around 80-90% confluent under the microscope.

**Step 2: *HeLa* cell passaging**

1. Mix DMEM with pen-strep and FBS to final concentration of 10% FBS and 2% penicillin-streptomycin. Then warm the DMEM mix, 1X PBS buffer, and TrypIE at 37 °C for around 1 hour.
2. Remove the old media from the plates. Add 5 mL 1X PBS buffer to the plate from the edge and shake gently on the bench, then remove the PBS using pipette.
3. Add 2.5 mL of TrypIE and incubate at 37 °C for around 10 minutes to dissociate the cells from the plate floor.
4. After 10-min TrypIE incubation, quench the reaction by adding 7.5 mL DMEM mix by moving the plate gently on the bench.
5. To passage 1 plate to 3 plates, prepare the new plates by adding 6.7 mL DMEM to each plate. Write down the cell line name, the passage number, operator name, and date on the plates for record.
6. Add 3.3 mL of parent cells to each new plate. Mix gently on the bench, then incubate at 37 °C and 5% CO_2_ for 2 days.
7. Change the media every two days by following steps 2-1 and 2-2, then add 10 mL warm DMEM mix to the plate.

**Step 3: *HeLa* cell harvest**

1. Remove old media from the plates with a pipette from the edge of the plate. Add 5 mL 1X PBS buffer (ice cold, pH = 7.4) to the plate from the edge and shake gently on the bench. Remove the PBS using pipette. Repeat this step 2 additional times.
2. Add 2 mL 1X PBS buffer (ice cold, pH = 7.4) to each plate and scratch the cells off the plates into 50 mL falcon tubes.
3. Pellet and collect the cells through centrifugation at 4,200 rpm and 4 °C for 20 minutes.
4. Add 20 mL 1X PBS buffer (ice cold, pH = 7.4) to wash the cells again. Repeat this step 2 additional times.
5. Centrifuge the cells down at 2,000 rpm and room temperature for 5 minutes. Remove the supernatant and store the cells at -80 °C for future recovery or lysis.

**Step 4: *HeLa* cell lysis**

1. Resuspend *HeLa* cells into the lysis buffer (1 μM PMSF and 20 mM NaF in PBS, pH = 7.4).
2. Homogenize cells in a sonicator for 5 minutes and allow to rest for 5 minutes, repeat this step two additional times.
3. Centrifuge the cell lysate at 15, 000 ×g, 4 °C for 30 minutes.
4. Evaluate protein concentration and patter using Pierce™ BCA Protein Assay Kit and SDS-PAGE. Aliquot *HeLa* protein samples and store at -80 °C for future use.

# **Python coding for reporter ions extraction for quantification**

check all codes and files at: <https://github.com/dahangyu/Protein-level-TMT-quantification>

# **Reference:**

(1) Yu, D.; Wang, Z.; Cupp-Sutton, K. A.; Liu, X.; Wu, S. Deep Intact Proteoform Characterization in Human Cell Lysate Using High-PH and Low-PH Reversed-Phase Liquid Chromatography. *J. Am. Soc. Mass Spectrom.* **2019**, *30* (12), 2502–2513. https://doi.org/10.1007/s13361-019-02315-2.

(2) Guo, Y.; Yu, D.; Cupp-Sutton, K. A.; Liu, X.; Wu, S. Optimization of Protein-Level Tandem Mass Tag (TMT) Labeling Conditions in Complex Samples with Top-down Proteomics. *Analytica Chimica Acta* **2022**, *1221*, 340037. https://doi.org/10.1016/j.aca.2022.340037.
